# Arrata

**Published:** 1849-08

**Authors:** 


### CONTENTS OF THE AUGUST NUMBER.

ORIGINAL COMMUNICATIONS. Page.

Art. I.—Report on the Diagnosis of Epidemic Cholera, made to the Buffalo Medical Association . . . . . . . . . . . . . 121
Art. II.—Muscular Rheumatism, as connected with Atmospheric Electricity. by N. B. Sherwood, M. D. . . . . . . . . . . . . 128
Art. III.—Tetanus produced by the application of Nitrate of Silver to diseased rectum, by John K. Hardenbrook, M. D. . . . . . . . . . 133
Art. IV.—Cases of Hydrocephalus successfully treated by Iodide of Potassium, by William Gardener, M. D. . . . . . . . . . . . 138
Art. V.—Buffalo Hospital of the Sisters of Charity: Surgical department. Report of Cases of Fracture, &c., by Dr. Franklin H. Hamilton. . . . . 140
Art. VI.—Remarks on manual delivery of the Plocenta, by R. A. Richardson, M.D. 145
Art. VII.—Case of Purpura Hœmorrhagica, by S. C. Rogers, M. D. . . . 146

ECLECTIC DEPARTMENT.

On Influenza and Ozone, by Dr. Spengler . . . . . . . . . 148
Ozone, aud its connection with Epidemic Diseases . . . . . . 149
Epidemic Dysentery successfully treated by Nux Vomica, by W. M. Cornell, M. D. 150
Camphorated Chloroform Liniment. . . . . . . . . . . 150
Ohio State Medical Convention. . . . . . . . . . . 151
Treatment of Epidemic Cholera, by Lewis Shanks, M. D. . . . . 155
Cholera, by M. Ward, M. D. . . . . . . . . . 160
On the Influence upon health, on the introduction of Tea and Coffee, &c., by S. Jackson, M. D. . . . . . . . . . . . . 161
Nitrate of Silver as a local application in Erysipelas, by Andrew E. Budd, M. D. 168
On the Fluid Secretions in Cholera, by M. Mialhe. . . . . . 171

EDITORIAL DEPARTMENT.

Deaths of Physicians by Cholera . . . . . . . . . 173
Microscopical appearances in the Intestinal discharges and muscular fibres of a patient who died of epidemic cholera . . . . . . . 173
Cholera at Buffalo aud the West. . . . . . . . . 174
M. Gunn, M. D. . . . . . . . . . . . 175
Miss Blackwall, M. D. . . . . . . . . . . 175
A practical Treatise on the Domestic Management and most important Diseases of advanced Life, by G. E. Day, M. D. . . . . . . . . 176
A manual of Auscultation and Percussion, by M. Barth. . . . . 176
The Casket and the Ribbon, by W. H. Gwinelle, M. D. . . . . 177
Southern Medical Reports . . . . . . . . . . 178
Annalist . . . . . . . . . . . . . 179
Annual meeting of the Medical Association of Southern Central New York . 179
State Medical Association of Illinois. . . . . . . . 180
Institution for Imbeciles and Idiots . . . . . . . . 181
Sulphur and Charcoal in Cholera . . . . . . . . 182
Tribute to the Medical Profession. . . . . . . . . 183

